# Book Reviews

**Published:** 1832-08

**Authors:** 


### CRITICAL ANALYSES.

**Quæ laudanda forent, et quæ culpanda, vicissim,**

**Illa prius, cretâ; mox hæc, carbone, *notamus.*—Persius.**

*An Introduction to the Practice of Midwifery.* By the late THOMAS DENMAN, M.D., Licentiate in Midwifery of the College of Physicians, London; and Honorary Member of the Royal Medical Society at Edinburgh. *Seventh Edition, with a Biographical Sketch of the Author. With additional Modern Information on the various Subjects treated of in the Work; and a Dissertation on the Transfusion of Blood in the more dangerous Varieties of Uterine Hemorrhage.* By CHARLES WALLER, M.D., Consulting Accoucheur to the London and Southwark Midwifery Institution; Lecturer on Midwifery and Diseases of Women and Children, &c.—8vo. pp. 525. E. Cox, London.

LABOUR cannot be more usefully bestowed, nor talent more usefully exerted, than in bringing up the standard works of the best professional writers to the improvements of the day. When Dr. DENMAN first published his work, no other could compete with it; and even now, in its original form, it is one which might safely be taken as a guide for the young practitioner and student in midwifery. In some parts, indeed, additions were required, and in others the doctrines laid down admitted of modification and improvement; and from the proofs Dr. WALLER has given us, upon more than one occasion, of his practical ability, and his facility of imparting to others that which he knows himself, we consider him particularly well qualified for the rather delicate task he has undertaken.

We have no intention to enter into a formal review of Dr. Denman's work, with the contents of which every member of the profession engaged in midwifery is certainly acquainted. We shall confine ourselves entirely to a notice of those parts which have been enlarged or improved by the present editor, premising that his object has been to avoid speculative hypotheses and vague theories, and to substitute in their place that which is sound and practically useful.

The editor is aware that objections have been made, and he confesses not without some reason, to the arrangement of some of the author's subjects; and he has not substituted any other, from the apprehension that it might be considered too great an alteration of a work which has been so long and so justly celebrated. He has, therefore, followed the original text, making what observations he thought necessary at the end of each particular paragraph. In order to distinguish these, they have been printed in a smaller type and enclosed between brackets. Dr. Waller's additions to the original work will be found chiefly in the articles on Transfusion; Instruments for the worst forms of Prolapsus; Puerperal Fever; and Diseases of the Genital Organs. Upon many other subjects he has also, but more briefly, pointed out the improvements that have been suggested since the time that Dr. Denman wrote.

Practical hints are always valuable, and therefore we shall select some which are given by Dr. Waller. The first we notice refers to a very troublesome, although not dangerous affection of females, pruritus of the external parts of generation. Dr. W. says that the local application which, in his experience, has produced the greatest degree of relief, is a solution of the nitrate of silver, beginning with five grains dissolved in an ounce of distilled water, and gradually increasing it to ten grains: "when the itching is caused by little herpetic eruptions situated on the inner surface of the labium, the Argent. Nit. seldom fails in giving relief."

Upon inflammation of the unimpregnated uterus, Dr. W. has added some useful observations, and to him also we owe the following practical remarks.

"Inflammation sometimes attacks the cervix uteri only, the other parts remaining perfectly free from disease; the pain experienced is much less than in the former variety of inflammation, and is not aggravated by pressure from without: if an internal examination be made, the os uteri will be found somewhat open and its lips tumid, and, when pressed upon, the patient will complain of pain. There is a considerable quantity of thick white mucus voided, and occasionally blood is discharged. The proper treatment at first consists in the application of leeches to the vulva, attention to the state of the bowels, and the injection of some anodyne fomentation up the vagina. In order to procure sleep, an opiate should be administered at bedtime, and it will be found useful to combine it with a diaphoretic, as perspiration usually affords relief. A chronic thickening of the part is frequently the result of this inflammation; mercury given so as to affect the mouth will be found the best remedy in these cases, especially if combined with the extract of hemlock; three or four grains of the latter with one grain of calomel should be taken three times a day. The editor has witnessed several cases in which impregnation took place with this state of parts: as might be expected, very great pain was experienced, and a long period of time required before dilatation could take place at the time of labour. Does this thickening of parts ever lay the foundation of scirrhous disease? The editor is inclined to think it sometimes does.

"The os uteri is subject to the formation of a very peculiar growth or tumor, made of granulated masses of different sizes; it has been called the cauliflower excrescence, from its fancied resemblance to the vegetable of that name. It is an incurable disease, but if attended to early, and proper measures be had recourse to, the patient's life may be prolonged and her comforts increased. From the surface of the tumor a limpid fluid resembling water is secreted, which is discharged from the vagina, and this forms the most prominent symptom of the complaint. As the disease proceeds, hemorrhage occurs, and very frequently to an alarming extent, in consequence of the giving way of the vessels which compose the bulk of the tumor.

"The growth of the tumor is in some instances slow, in others more rapid, depending greatly upon the degree of resistance offered by the vagina, whether it be firm and rigid, or soft and yielding; and from this circumstance the cause may be explained why the disease extends more rapidly in married females, especially if they have borne children. If a female become pregnant during the progress of this disease, and the medical man has not an opportunity of examining before labour commences, he is very likely at first to confound it with a presentation of the placenta: there will be a very copious discharge of blood, and there will be a substance in the vagina, granular to the feel; on making a critical examination, however, the cauliflower excrescence will be found growing from the os uteri; whereas the placenta is contained within it.

"The object to be attained in the treatment, is that of retarding the growth of the excrescence, which is best accomplished by lowering the force of the circulation, by abstracting blood, where the patient's constitution will bear it, either by venesection from the arm, or by cupping from the loins. The bowels should be kept in a soluble state by means of ol. ricini, or any other mild laxative, and the patient placed upon rather a spare diet, great care being taken to avoid any thing of a stimulating nature; the injection of astringent solutions into the vagina has also been recommended with a view of lessening the tumor by diminishing the size of its blood-vessels, and also with the intention of increasing the tone and firmness of the vagina, by which a natural impediment will be offered to its future enlargement. The horizontal posture should be kept to, and if the female be married, she should be recommended to have a separate bed from her husband. If the practitioner be called in early, he will by these means frequently be enabled to retard the progress of the disease, although it is one which is in its nature incurable." (P. 75.)

Vomiting during pregnancy, however violent it may be, is but rarely attended with danger; but the practitioner is often applied to, to relieve so troublesome a symptom, and we fear he seldom succeeds in satisfying his patient of the efficacy of any of the remedies that are usually employed. Dr. W. suggests the use of hydrocyanic acid,* beginning with two minims, and increasing it to four minims: this dose may be given every four hours for three or four times, and afterwards waiting for a time, and, if necessary, recommencing it. "In several very obstinate cases the editor has succeeded with this medicine after other means had failed."

*Detection of Pregnancy by the state of the Urine.* A few months ago, M. Nauche published, in the "Lancette Française," an account of some experiments on the urine of pregnant women.

* Scheele's strength.

He states that, by allowing the urine of pregnant women or nurses to stand for some time, as from thirty to forty hours, a deposit takes place of white, flaky, pulverulent, grumous matter, being the caseum or peculiar principle of the milk formed in the breasts during gestation. This interesting subject has not escaped Dr. Waller's attention; and he observes, that the test would have been invaluable had subsequent trials proved its efficacy, but this has not been the case.

"At the request of the editor, Mr. Pereira, the chemical lecturer at the General Dispensary, has investigated the subject, and he thus reports: 'I have examined the urine of several pregnant women, and although, when far advanced in pregnancy, the urine, on standing, deposited one or more thin, delicate, bluish-white flakes, apparently of caseum or coagulated albumen, yet, in the early months, I have not invariably found this appearance. As a still farther proof of the insufficiency of this sign, as a test for pregnancy, I may add that, where the caseum is found in the urine, it is not to be regarded as an absolute proof of pregnancy. Leopold Gmelin, in his *Handbuch der Theoretischen Chemie, 2ten bandes, 2te abtheilung*, p. 1417, mentions, on the authority of Caballe, that caseum was found in the urine of a healthy young widow. The same writer also states, on the authority of Wurzer, that caseum was found in the urine of a man, whose breasts were previously swollen." (P. 171.)

Dr. W. makes some good practical remarks on uterine hemorrhage, in addition to those of Dr. Denman, and he describes very carefully the mode of performing transfusion: the instruments required are also represented in a plate.

*Spontaneous Inversion of the Uterus.* "Some persons have doubted the possibility of a spontaneous inversion of the uterus ever having taken place, and the editor confesses that he himself was formerly one of this number; but a case related to him by his friend Dr. Williams, of Guilford street, has quite convinced him that such an occurrence occasionally takes place. The Dr. had attended a lady in her fourth labour; the pelvis was of ample dimensions, the child soon expelled. The funis was tied and the child separated: immediately afterwards there was a long expulsatory pain, by which Dr. W. naturally enough inferred that he should find the placenta detached and thrown off. On regaining his seat by the side of the bed, and making an examination, he felt a large substance protruding from the vagina, which proved to be the uterus in an inverted state. The organ, with the placenta still adhering, was promptly returned to its proper situation, and every thing went on favorably. This case affords a clear instance of inversion taking place from the action of the uterus itself, as it took place whilst the Dr. was engaged with the child, not the slightest extension having been made by the cord: in fact, it had not been touched by the hand.

"In cases of chronic inversion, the protruding portion of the uterus has several times been removed by the application of a ligature, composed either of wire or any other substance of sufficient firmness. This operation has been attended with perfect success, and unaccompanied with any dangerous symptoms. For further information on this subject, the reader is referred to an essay, published many years ago by Mr. Newnham, a very intelligent surgeon residing at Farnham in Surrey." (P. 424.)

Dr. Waller very properly objects to Dr. Denman's mode of classing different affections of the abdomen under the same term, puerperal fever. In order to explain his own views on this important subject, he submits the following observations from his Elements of Practical Midwifery, second edition, p. 157 et seq.

"Most of the complaints which attend the puerperal state are of a highly active character, and require therefore great activity in their treatment; they are attended not only with increased action, but with a corresponding degree of power; on the other hand, it sometimes happens that, although action may be in excess, power may be rapidly on the decline, and hence, in the curative means, care must be taken not to employ remedial agents which have a tendency still further to lessen the powers of the system. The abstract nature of power and action is unknown, but the distinction between the two is highly important in practice: the balance between them is maintained during health, but this balance may be destroyed by any of the remote causes of disease: some of them are directly stimulant; but many, perhaps most, have at first a tendency to depress, so that the high action which follows is not directly produced by the application of the cause, but in consequence of the reaction which follows.

"The want of a just discrimination between power and action, has been productive of much of the perplexity and diversity of opinion which has prevailed respecting the disease called puerperal fever: it will be found, on consulting the works of different authors, that remedies the most diametrically opposite in their nature, have been proposed, and successfully adopted, for the cure of an affection which they designate by the same name. It is, however, quite inconsistent to suppose that a disease of the same character can be cured by bleeding and antiphlogistic measures on the one hand, and by stimulation and support on the other; if one plan be right, the other must be decidedly wrong: and yet these authors refer to cases occurring in their own practice as proofs of the superiority of their particular treatment. The simple matter of fact is that the diseases described by these writers are essentially different; they assume quite an opposite type: viz. in the one there has been action with power, in the other action without power: the one has been acute inflammation of the peritoneum attended with its usual symptoms, and requiring its usual treatment; the other has been the low or passive form of inflammation, attended with, or perhaps depending upon, fever of the adynamic or typhoid type; and it is to this latter form of complaint that the term puerperal fever will be applied in the present essay, whilst the former variety will be designated by the name of peritonitis.

"The author is fully aware that there are cases of a mixed character, which cannot be strictly classed under either of these heads, as they appear to partake somewhat of the nature of both; cases, for example, in which a certain degree of power is present, requiring antiphlogistic remedies to a certain extent, where probably, at the very onset, a small bleeding from the arm might be required, but where inevitable destruction would follow that free use of the lancet which is so peremptorily demanded in the more acute attacks of peritonitis; he is nevertheless of opinion, if the attention of the student be directed to the distinguishing characters of the two forms of disease, that experience and his own good sense will enable him to discriminate those varieties in which the treatment requires to be modified, and will therefore lead him to adapt his remedies to them. It has been too much the fashion to class nearly all the affections to which the abdomen is exposed at the time of child-birth under the one head Puerperal Fever; and, in consequence of this circumstance and the varieties of treatment recommended, great inconvenience and difficulty have been experienced by the young practitioner in making up his mind as to the proper mode of proceeding." (P. 494.)

The necessity of discriminating between the two diseases, peritonitis and puerperal fever, is forcibly insisted upon by Dr. W., and a table is given exhibiting the leading marks of distinction between them.

In a work like the present it would be unreasonable to expect a detailed account of the pathological information that has recently been elicited by DANCE, TONNELLÉ,* R. LEE, &c. respecting puerperal fever as dependant upon inflammation and suppuration of the uterine veins and lymphatics; but we think Dr. Waller should not altogether have passed over this important subject. It is replete with interest and instruction, and the student ought certainly to be made acquainted with at least the substance of the facts and doctrines to which we refer. We observe the same deficiency also upon the subject of phlegmasia dolens: the pathological researches of VELPEAU, DANCE, BOUILLAUD, and R. LEE, are not mentioned, although here the editor had a good opportunity of adding much important matter to the *now* imperfect chapter of Dr. Denman.

These omissions should be filled up in a future edition, which we doubt not will speedily be required. The work was always in great demand, and it will certainly be much more popular now that Dr. Waller has so materially contributed, by his edition of it, to render it additionally worthy the attention of obstetric students and practitioners.

* Vide London Med. and Phys. Journal for July 1830, and several succeeding numbers.

*A Treatise on the Injuries, the Diseases, and the Distortions of the Spine; founded on an Essay to which the Jacksonian Prize, for the Year* 1826, *was adjudged by the Royal College of Surgeons.* By R. A. Stafford, Member of the Royal College of Surgeons; Surgeon to the St. Mary-le-bone Infirmary; and formerly House-Surgeon to St. Bartholomew's Hospital.—8vo. pp. 302. Longman and Co., London.

If medical writers in general followed the example of Mr. Stafford, whose object is to "state the facts which have come under his own observation, rather than to relate the opinions of others," our literary catalogue would be less in bulk, but richer in materials than it is at present. But the fact is, that few authors have much to say from their own personal experience: a book, however, must be written, and, as the real resources of the writer are too scanty to eke out a respectable quantity of pages, he is driven to draw largely upon the information which others have already bestowed, and the principal skill that is manifested too often consists in the dexterity with which he appropriates to himself that to which he has really no claim whatever; a good compilation upon any important practical subject must always be welcome to the profession, and creditable to the author; but it should bear its proper name, and not be ushered into the world under the (in every sense of the word) imposing title of an "original essay:" we are led to make these remarks from having before us at the moment several of these "originals," in which, in truth, there is not one spark of originality, and which, if ever looked over by the pilferers themselves, must suggest to them the truth of the line of Martial, "Dicitque mihi mea pagina, fur es." Mr. Stafford has had neither leisure nor inclination to make a mere compilation, nor even to enter into long disquisitions on subjects which, from the imperfect knowledge we at present possess of them, could only terminate in doubt: he has obtained his information chiefly from the bedside of the patient, the examination of the diseased parts after death, or from morbid preparations. For the arrangement of the work he is not responsible, as he has followed that proposed by the Jacksonian committee of the College. He first treats of the congenital diseases of the spine; then the injuries; the diseases and distortions of the vertebræ; and, lastly, on those of the medulla and its membranes. Mr. S. has endeavoured to compress and simplify the matter as much as possible, confining himself only to the most important diseases affecting the spine.

*Spina Bifida* is first considered. This malformation was first observed by the Arabian physicians, who, from their ignorance of its real nature, believed it to arise from a double formation of the spinous processes, and hence the term now generally employed. Mr. Stafford correctly states that, from the numerous dissections which have been since made of this disease, it has been discovered that it is owing to a deficiency of the spinous processes at the rings of the vertebræ. The membranes of the medulla are deprived of their bony covering at one particular part, and consequently, not having their natural support, become thrust through the opening by the preternatural secretion of fluid contained in the canal. Thus a pouch or tumor is formed upon the spine, which by SAUVAGES was denominated *hydrorachitis;* by PINEL, *hydrorachis,** and by FRANK, *hydrorachia?*† The usual symptoms which attend upon spina bifida are paralysis of the lower extremities, with the power of motion and sensation more or less deficient, want of power to retain the fæces and urine, frequent diarrhœa, deformed limbs, convulsions, emaciation, or general weakness. Mr. Stafford states, however, and we can confirm the fact from our own observation in these cases, that there is no one of these symptoms which is invariably present. He mentions two instances in point. In one, the child is five years old, and is as stout and healthy as any child of its age; and in the other, although it died, the motion and sensation were as perfect as natural, and no other defect or bad symptom was present. Children labouring under this formidable, and, with few exceptions, incurable disease, are usually destroyed by it at a very early age. The tumor gradually increases in size, and at length it bursts, from ulceration or gangrene of its walls; its contents are evacuated, and death terminates the existence of the patient. The period of time at which this event occurs is uncertain. Cases have been related where the subjects of this disease have lived much beyond the period of infancy, from twenty to thirty, and even to as advanced an age as fifty. Mr. STANLEY mentions a case in his Lectures, in which the person is in his twenty-second year. In these cases the tumor remains stationary, neither increasing nor diminishing, and its walls become thickened, firm, and opaque.

Spina bifida is usually described as a lumbar tumor, "but it may occur in any part of the spinal column, from the cervical portion to that of the sacrum; and various authors relate well authenticated cases where each part, and sometimes the whole of the canal has been defective and involved in the disease."

Even in the most favorable cases of this disease a happy termination cannot be expected. It is true, as Mr. Stafford observes, that a few cases have been related where the palliative treatment has succeeded in retarding and giving temporary relief to the disease, and where the consolidation of the parts over the malformation of the vertebral canal has taken place by the radical treatment of it, so that a kind of cure has been effected. "Spina bifida also is so frequently combined with some other disease or malformation, that it is but seldom that an opportunity is afforded us of relieving it; and even during the process, when there is a prospect of cure, there is so much danger from inflammation of the parietes of the tumor, or the medulla spinalis, that in many instances the patients have

* Nosog. Phil. † De Cur. Hom. Morb.

been known to die from one of these causes while under treatment." The ancient surgeons, regarding the disease as beyond their art, used only palliative measures; the moderns have proposed ligatures, setons, compression, and puncture. "The only methods which have been employed with any real advantage are compression and puncture: and for the introduction of both of these we are indebted to Mr. ABERNETHY: Sir ASTLEY COOPER,* however, was the first who had the good fortune to carry them into effect." Mr. Abernethy proposed applying gentle pressure upon the tumor from its commencement, with the idea of giving that support to the dura mater which it had lost from the deficiency of its bony covering, and at the same time of producing, if possible, the absorption of the fluid. He himself had never an opportunity of trying this method, but Sir A. Cooper employed it in the following case, which is quoted by Mr. Stafford.

"'James Applebee, Baldwin street, Old street, was born on the 19th of May, 1807; and his mother, immediately after his birth, observed a round transparent tumor on the loins, of the size of a large walnut. On the 22d of June, 1807, the child was brought to my house, and I found that, although it had spina bifida, the head was not unusually large; and the motions of its legs were perfect; and its stools and urine were discharged naturally. I applied a roller around the child's waist, so as to compress the tumor, being induced to do so from considering it as a species of hernia, and that the deficiency of the spine might be compensated for by external pressure. The pressure, made by the roller, had no unpleasant influence on its voluntary powers; its stools and urine continued to be properly discharged; but the mother thought that the child was occasionally convulsed. At the end of a week, a piece of plaster of Paris, somewhat hollowed, and that hollow partly filled with a piece of loose lint, was placed upon the surface of the tumor: a strap of adhesive plaster was applied, to prevent its changing its situation; and a roller was carried around the waist, to bind the plaster of Paris firmly upon the back, and to compress the tumor as much as the child could bear. This treatment was continued until the month of October, during which time the tumor was examined about three times a week, and the mother reported, that the child was occasionally convulsed. When the child was five months old, a truss was applied, similar in form to that which I sometimes use for umbilical hernia in children, and this has been continued ever since. At the age of fifteen months it began to make use of its limbs; it could crawl along a passage and up two pair o. stairs. At eighteen months, by some accident, the truss slipped from the tumor, which had become the size of a small orange, and the mother observed, when it was reduced, that the child appeared in some degree dull; and this was always the case, if the truss was left off for a few minutes, and then reapplied. At fifteen months

* Surgical and Physiological Essays, Part iii. p. 134, 1797.

he began to talk; and, at two years of age, he could walk alone. He now goes to school, runs, jumps, and plays about, as other children. His powers of mind do not appear to differ from those of other children. His memory is retentive, and he learns with facility. He had the measles and smallpox in the first year, and the hooping-cough at three years. His head, previously and subsequently to the bones closing, has preserved a due proportion to other parts of the body. The tumor is kept by the truss entirely within the channel of the spine; but when the truss is removed, it soon becomes of the size of half a small orange. It is therefore necessary that the use of the truss should be continued. When the truss is removed, the finger can be readily pressed through the tumor into the channel of the spine.'

"In this case, although simple pressure succeeded, yet it might not be attended with equal advantage in all, as symptoms of compression of the brain, or some other bad effect, might be produced. We may observe, also, that this method of treatment does not effect a permanent cure, but is only, as Sir Astley Cooper very properly termed it, palliative; for as soon as the truss is removed the sac of the tumor fills again, and becomes as large as before. Its parietes, likewise, do not increase to that degree of thickness as to form a permanent support to the medulla spinalis; and therefore, as there is no consolidation of the parts, we cannot expect a radical cure." (P. 30.)

In Mr. Stafford's opinion, the most effectual, and perhaps the only possible way of performing a radical cure of spina bifida is by frequently puncturing the tumor, making gentle pressure upon it, and healing up the wound so as to prevent the admission of air, as recommended by Mr. Abernethy. We have ourselves tried this plan in two cases, in both of which the children were free from any other malformation or disease: they were, indeed, remarkably robust and active. The age of one child was four years, the other three years. In each case, the tumor was frequently punctured with a fine needle, and its contents evacuated. No inflammation or unpleasant symptom arose from the puncture, but the tumor speedily refilled, and became as large as before. We could not devise any means by which pressure could be effectually applied to the part. Bandages were applied in various ways, without avail: a part of an India-rubber bottle was also applied, after the contents of the tumor had been evacuated, and bound firmly down. A mould of plaster of Paris was made over the tumor and strapped down with adhesive plaster, with as little success: the tumor, in spite of any of these means, quickly regained its former magnitude, and raised up whatever was placed over it. We have long lost sight of these children, and know not the subsequent progress of the disease. In each case, the tumor was gradually increasing, was at last large enough to contain about two ounces of fluid, and the parietes were so thin as to be nearly diaphonous. We should observe, that in one of these examples the child appeared restless and uncomfortable for a short time, and the pupils became dilated when even moderate pressure was applied.

Mr. Stafford relates an interesting case, which fell under his own observation, which well illustrates both the progress and the process by which the radical cure of spina bifida is effected. The whole of the chapter upon this subject is worthy of attention, as it contains a concise and clear account of the most important facts that as yet have been promulgated, both in relation to the nature of the disease and the various modes of treatment that have been suggested.

*Injuries of the Spine.* In the second and third chapters, Mr. S. treats upon *concussion, and fractures and dislocations of the spine;* and the various cases he gives are particularly valuable, as illustrating the precise effects resulting from injuries inflicted on different portions of the spinal canal.

In concussion of the spine, the inferior extremities of the body are usually alone paralysed, but sometimes the superior parts suffer more or less from it, although the blow may have been inflicted on the vertebral column beneath that portion of the medulla from whence they receive their supply of nerves.

"Thus, for instance, the middle of the back may have been struck, and yet the nerves, supplying the upper extremities, as well as those supplying the lower limbs, may lose their power. Such cases are not uncommon, and the following is one which occurs to my recollection: A man received a violent kick from a horse on the most projecting point of the dorsal vertebræ; he instantly became paralysed in the lower extremities, and the arms likewise lost all power of motion, and partially that of sensation. Here the blow was received below where the nerves arise which supply the arms, and thus the superior extremities became paralysed rather from the general shock than from any injury done to the medulla immediately at that part. When, however, the upper extremities are influenced by the shock, they generally suffer in a much less degree than the lower, only perhaps partially losing their motion or sensation, and this may occur in one or in both of them at the same time; they usually, also, recover their powers much more quickly. It does not always follow, however, after a blow has been received on the spine, that all the parts below shall become paralysed. Sometimes only the muscles suffer which are supplied by nerves from the injured part, as was the case in the following instance: A pack of goods fell upon a man who was brought to St. Bartholomew's Hospital, which had struck him between the scapulæ, at about the seventh cervical and first and second dorsal vertebræ: his arms became immediately and totally paralysed, and there was a partial loss of power in the muscles of respiration. Neither the inferior extremities, the bladder, nor the rectum, were in the least degree affected. It is probable, in this case, that the origins only of the nerves which supplied the arms were injured." (P. 59.)

Injuries of the spine cause other anomalous symptoms, as may be learnt from the following case.

"October 1831. About eight years from the above date a man, belonging to the town of Penkridge, in Staffordshire, fell from the top of a waggon-load of hay. He was taken up in a perfectly helpless state, and was immediately carried to bed; he had struck his back upon the second, third, and fourth lumbar vertebræ, which were considerably displaced laterally, the body leaning to the right side, leaving but little doubt that the spine at that part had suffered fracture. He was perfectly paralysed below the injury; the fæces escaped involuntarily, and the bladder could not expel its contents; the arms likewise were partially paralysed, in both the powers of feeling and motion. The treatment of the case I am unacquainted with, but he has kept his bed ever since, and his present state is as follows: The muscles of the right arm are so contracted that it is closely fixed to the side; the forearm, from the same cause, rests upon the humeral part; the wrist is bent on the forearm, and the fingers are firmly clenched in the palm of the hand: the sense of feeling also is partially lost; the left arm is affected in the same manner, but not in so great a degree; the right leg has both the power of motion and feeling; the left leg has the power of feeling, but not that of motion; the sphincter muscle of the rectum remains paralysed, the fæces still escaping involuntarily, and the bladder only expelling half its contents.

"The various symptoms just related are certainly very extraordinary, and, had it not been for the discoveries of modern physiologists, they could not have been accounted for; and, even as it is, some of them are still involved in great obscurity. For example, how can we for certain explain why, in one case, the arms alone should be paralysed; in another, only the bladder and rectum, and one leg partially; and in a third, that the parts above the place where the blow was received should suffer as well as those below? Such phenomena cannnot be satisfactorily accounted for, and the only attempt at elucidation we can offer is, that the origins of those nerves, or that particular part of the substance of the medulla with which they are connected, supplying the parts affected, have more particularly suffered." (P. 60,)

Since the discoveries of Sir Charles Bell, and Magendie, the cause of the unequal diminution of the power of motion and sense of feeling in concussion of the spine can, however, be more easily explained. They have indisputably proved that the anterior and posterior roots of the spinal nerves possess two different functions: the anterior that of motion, and the posterior that of sensation. It has been proved by experiment, that, if the anterior roots of the nerves of the spinal chord be divided, the power of motion of the muscles which they supply is entirely lost; and if the posterior roots be severed, the sense of feeling no longer remains. These facts will explain many of the symptoms which are met with in injuries and diseases of the spine, which before could not be understood.*

Mr. Stafford briefly but accurately describes the symptoms and treatment of concussion of the spine, and relates two interesting cases which occurred at St. Bartholomew's.

Fractures of the spine are attended by the same symptoms as those of concussion, excepting that they are usually much more severe, and remain so until death. The progress is more unfavorable than in concussion, as it usually happens that some irreparable mischief is done to the medulla or its membranes. "There is no reason, however, why, in some cases of simple fracture, the patients should not ultimately recover; as, when there is no displacement of the bone, the injury amounts to very little more than concussion." As a proof that fractures of the spinous processes frequently get well, two cases are related. Mr. Stafford mentions two preparations which shew that the vertebræ may unite after fracture; one of these specimens is in the College of Surgeons, and the other belonged to the museum of our anatomical preceptor Mr. Brookes.

Mr. S. has not met with any cases recorded of reparation of laceration of the medulla, nor has he seen any preparations illustrative of this fact. "Wounds of the medulla, however, have been known to get well. M. Ollivier has related several cases where the spinal cord has been wounded by the point of a sword, and the patients have either wholly or partially recovered?

"In one instance, a young man was wounded by a quadrangular poniard on the left side of the neck, just below the ear, and at the origin of the spinal cord. He immediately lost all power of motion and sensation in all the parts below the head; in fact, he was per-

* In vol. iii. of the Med.-Chir. Transactions, a curious case of loss of sensation in the upper and lower extremities, independent of paralysis, is related by Dr. Yelloly. The subject of it, a man, after being much heated and fatigued, slept with the window open. In the morning the feet and ankles were numb, without pain, and *without the muscular powers* being at all affected. A tingling in the little finger afterwards occurred, and gradually both hands were, in a considerable degree, insensible. The hands up to the wrist, and the feet halfway up to the leg, were perfectly insensible to any species of injury, as cutting, burning, &c. The insensibility does not suddenly terminate; it exists to a certain degree nearly up to the elbow, and for some distance above the knee. He accidentally put one of his feet into boiling water, but was no otherwise affected by it, or aware of the high temperature, than by finding the whole surface a complete blister. The extremities were insensible to electricity. The relater of this interesting case observes, "that the existence of muscular power, and the faculty of directing its exercise by the will, when the nerves have entirely lost that sensibility which is always regarded as necessary to the conveyance of volition from the sensorium, are circumstances apparently irreconcilable with any knowledge which we at present possess of the mechanism by which the will acts in the production of voluntary motion." The experimental researches of the eminent physiologists above mentioned satisfactorily explain this circumstance.—Editors.

fectly paralysed. At the eighteenth day he began to recover a little feeling in the left side of the body. On the twentieth day he could move slightly the fingers and toes of the arm and leg of the same side, and he continued improving until the thirtieth day, when sensation began to return in the right side, being gradually followed by the return of the power of movement. He received the injury on the 31st of January, and could walk, though slowly and feebly like a child, on the 26th of May following. M. Ollivier also records other cases, where the patients either wholly or partially recovered after the medulla had received a wound from a clean cutting instrument. It appears, therefore, that if the medulla be only cut or pricked, the wound will heal most probably by the first intention; but, if it be lacerated, there is as yet no fact to prove that such an injury to it will be repaired." (P. 93.)

The general treatment of fracture of the spine is the same as that in concussion, but it is more imperatively necessary that the parts should be kept quiet. The two following cases shew how important it is that this caution should be borne in mind.

"Case I. July 25th. A man having received a kick from a horse, upon the back of his neck, became instantly paralysed in all parts of the body below the injury. He was immediately carried to St. Bartholomew's Hospital, and in about twelve hours after the accident delirium came on. The surgeon desired that the head might be shaved, in order that the proper remedies might be employed. In doing this the barber turned the head a little on one side: the patient instantly fell from under his hand, and expired. Upon examination it was found he had fractured the third cervical vertebra, and that the sharp point of the fracture had pressed upon and wounded the medulla.

"Case II. A man fell from a great height, and broke his neck. An officious nurse, in moving him up, as she thought to relieve him, let his head fall backward. He instantly expired. He had fractured the second cervical vertebra." (P. 94.)

In fractures of the spine, it often happens that the ring of one or more of the vertebræ is beat inwards in such a manner as to compress the medulla and its membranes. The experiments that have been performed on animals favor the opinion that in such instances the operation of trepanning might succeed. Mr. S. states that Baglivi and Pallisio have frequently trepanned the spine of dogs, and they have found in many instances that it did not appear to give rise to much suffering, or to affect the functions of the medulla, unless the dura mater was pricked, when convulsions were produced. Experience as yet, however, he says, on the human subject is rather against the operation. The spine, when a piece of fractured bone has been depressed upon the medulla, has been trepanned five times, and in all these cases the patients have died.

In the chapters above mentioned there are many instructive cases detailed, both of fracture and dislocation of the spine.

The sixth chapter treats of *Diseases of the Vertebræ from Rickets and various other causes.* We assent to the indications of cure laid down by Mr. Stafford in cases of rickets, but we are inclined to differ from him to a certain extent as to the mode by which the object may be attained. In many cases, exercise, both active and passive, both general and local, according to the strength of the patient, is the best therapeutic agent. We have seen many cases of rickets where all the ordinary means had been had recourse to ineffectually for a long time, and where exercise, judiciously enforced, with *occasional* recumbency, removed the tendency to disease, and gave solidity to the bones. Softening of the bones in rickets and Mollities ossium generally depends upon a defective assimilation of food, which is most commonly induced by excess of aliment and neglect of exercise. We may observe the frequent existence of rickets in the children of mechanics in large towns, and its comparative rarity in the children of cottagers in country districts; the former are usually much more liberally supplied with food of a nutritious quality than the latter, but these have the advantage which the others have not, of free exercise in the open air. It is true that Mr. Stafford admits the necessity and advantages of exercise in some cases of rickets, but, as a general principle of treatment, he relies more than we are inclined to do upon the effects of a constant recumbent position both at home and abroad; for he says that "when in the house the patient should lie on the sofa, or upon the outside of the bed, and, when out of doors, he should lie down, lying at full length, in a carriage constructed for the purpose."

The following subjects are next commented upon in a very satisfactory and practical manner. Mollities ossium; Hardening of their structure; Tumors formed in and pressing upon them; Fungus Hæmatodes; Abscess formed in the Substance of the Bone; secondary Symptoms of the Venereal Disease; Diseases of the Intervertebral Substances and of the Articulated Joints and Ligaments; Consequences of Diseases of the Vertebræ and their Cartilages; Angular Curvature and its effects; Lumbar Abscess.

Mr. Stafford divides the symptoms of diseases of the bones and cartilages into three stages. 1st. Those which occur before curvature. 2d. Those which are present while curvature is taking place. 3d. Those which happen during the progress of recovery.

"In the first stage, the patients generally feel great lassitude, look pale, and become emaciated without being able to assign any particular cause. They complain of weakness; of a dull heavy pain in the back at the point where the disease is present, and of a numbness and coldness in the lower extremities; their legs totter under them, and when they walk they frequently trip, and are incapable of placing the feet exactly where they wish to tread. They usually also express relief from being in the horizontal position, and complain that, when they rise next morning, the symptoms increase. They sometimes suffer extreme pain in the hip-joint, the knee, and the ankle, so much so that the malady is often mistook for a disease of one of these joints. They have cramps in the muscles of the lower limbs, and are often seized with convulsive twitchings, the result of the irritation caused by the diseased parts. A sense of tightness is often present at the stomach; pain is sometimes felt in the side, and exercise is fatiguing. They constantly feel chilly; the appetite fails them; the pulse becomes quickened, irritable, feeble, and occasionally irregular; in short, the health is generally deranged.

"As the symptoms advance into the second stage, and the curvature is forming, they become more or less paralysed below the diseased part; they gradually lose the power of the rectum, and of the bladder; the function of the intestines is diminished, by which costiveness ensues, and the urine cannot be expelled; the muscles of the legs and thighs became flaccid, and wasted, and by degrees lose both the power of sensation and motion: in this state they remain powerless, unless some favorable change in the disease takes place, until they die.

"The symptoms I have here related, are those which generally occur in a greater or less degree, but of course they vary according to the situation of the disease. If it be present in the neck, or in the upper dorsal vertebræ, then a greater part of the body is involved in the mischief; the respiration is affected, being rendered difficult and irregular, and the sense of tightness at the stomach and of the abdominal muscles is greater. If, however, it be situated lower down, the parts which receive their supply of nerves from the medulla beneath the disease, alone suffer a loss of power. It does not always follow, however, when the disease is seated high up in the neck, for instance, or on the superior dorsal vertebræ, that the muscles of respiration shall be affected. There is a case, under my care, at this present time, in St. Marylebone Infirmary, where all the cervical vertebræ are diseased (except the atlas and dentatus,) and there is an angular curvature, yet the respiration is in no way affected. The power of motion is perfect in the arms, but in the legs it is entirely lost, and the sphincter muscle of the rectum also is paralysed: a perfect sense of feeling remains throughout the whole body. The only way to account for such symptoms is, that the medulla is only partially compressed.

"During the progress of the disease, various symptoms occur, apparently unconnected with the spine, and this may be observed more particularly in the female. It frequently happens that a girl with a disease of the spine is attacked by all the symptoms of hysteria. One day she will be found apparently in perfect health and spirits, and the next to all appearance dying: she will be attacked by syncope, dyspnœa, &c. Sometimes, from the violent pain felt in different organs of the viscera, and from the constitutional symptoms accompanying them, it would seem that one of them was attacked by inflammation. Acute pain is frequently felt in the region of the liver, the stomach, and the intestines, and bearing all the characters of hepatitis, enteritis, peritonitis, &c. Sometimes there will be an attack of fever, resembling what is termed a continued fever. All these variety of symptoms I have witnessed. During the time I was house-surgeon to St. Bartholomew's Hospital, I recollect several instances of it. Out of half a dozen patients, one or more of them, perhaps, would be suffering from some of these symptoms, and it always appeared to me that they were greatly influenced by the state of the atmosphere." (P. 175.)

It is not denied that there is some difficulty in accounting for the symptoms just described: they are most probably, however, occasioned by the intimate connexion of the spinal nerves with the sympathetic. "Costiveness is induced by a disease of the spine, by which it is shewn that the coats of the intestines have a diminished function, and that the peristaltic motion is imperfect. From what can this arise, unless it is that the disease of the spine has affected the power of the sympathetic?" And again, in very severe injuries of the spine, we find that the intestines become instantly inflated with air, and that their functions are more or less paralysed. They are supplied by no nerve from the spine, but from the sympathetic alone.

"How can we explain so instant an effect, unless it is from the connexion of the spinal nerves with the sympathetic? If, then, it be allowed that the junction of the spinal nerves with the sympathetic has an influence over the functions of the intestines, why should it not have the same influence over other organs which are supplied by the same nerve? Should an increase of inflammation occur at the diseased part of the spine, is it not natural to suppose that it would more or less affect the nerves connected with it, and that secondarily, through its connexions with the sympathetic, the various organs supplied by this latter nerve may suffer? It appears to me, that, when we take a comprehensive view of the nervous communications of the medulla spinalis, with the sympathetic and other nerves, we cannot be surprised at any symptom that may be present. I am inclined to think, also, that the connexions just alluded to may account for some of the functions of the viscera, which at present are but imperfectly understood." (P. 180.)

The ninth chapter contains the Treatment of Diseases of the Bones and Intervertebral Substances.

*On the Distortions of the Spine.* The author commences this subject by observing that distortions of the spine arise from various causes, and it is therefore of the utmost importance, as relates to their treatment, that we should form an accurate diagnosis of their origin. Should they be produced from disease of the bones or intervertebral substances, the treatment employed for other deformities of the vertebral column would be highly injurious; and, *vice versâ*, should they be the result of rickets, or irregular actions of the muscles, the methods pursued for the relief of diseases of the bones would also be equally inefficacious. "Distortions of the spine are very rarely congenital; there is one specimen, however, in the Museum of St. Thomas's Hospital, where it is bent forwards in an infant who had spina bifida." Mr. S. regards it as a general law, that in all cases of contortion of the spine, a portion, more or less considerable, of the vertebræ in the concave side of the curvature is destroyed, either by ulceration, or by absorption from compression. At page 233 it is said, that in very bad cases of lateral curvature, the bodies of the vertebræ are so thrown out of their situations, that they are, as it were, rotated on the intervertebral substances, and, instead of their anterior surfaces looking forwards, they are turned laterally. "In some cases also the curvature is so great and acute, that paralysis is the result: this, however, does not commonly happen." We have never seen a case of paralysis from lateral curvature, nor do we believe in its existence without disease of the vertebræ, which is seldom present in lateral distortion. Mr. Stafford's general remarks upon the treatment of lateral curvatures are correct and clearly stated.

The eleventh and twelfth chapters treat briefly on diseases of the medulla and its membranes. The diseases of the contents of the spinal canal may be divided into two classes; those which affect the membranes, and those which affect the medulla itself. The former are liable to inflammation; to dropsy, or hydrorachitis; to extravasation of blood between them; to have cartilaginous depositions formed upon them; and to the formation of tubercles in their structure. All the membranes of the spine are liable to inflammation, which may seize idiopathically or from injury. The arachnoid is the one most frequently attacked by it, and when thus affected, it is called by some writers *Arachnitis Spinalis.*

"The cause of Arachnitis is very obscure. Sometimes it comes on very gradually, while at others suddenly. The symptoms which denote it are the following: the patient at first commonly finds himself generally unwell, without being able to describe any particular symptom, excepting that he has not the same power over his limbs as usual. At length he has pain in the back, in one particular part, or along the whole course of the spine. Contraction of the muscles of the back ensues, which varies from simple muscular rigidity to violent spasm, affecting one particular part, according as the disease is situated on its whole extent. Accompanied with these symptoms, there may be disury, retention of urine, and complete constipation. As the disease advances, the pain along the spine increases; the muscles become violently spasmed; there is difficulty of deglutition and respiration; convulsions of the whole frame; trismus; at length opisthotonos, and all the symptoms accompanying tetanus.

"The symptoms of arachnitis are not always equally acute. M. Ollivier remarks, that sometimes the patients only have pain and rigidity along the course of the spine, and are even enabled to walk or to turn themselves in bed almost until death. Neither are the symptoms in every instance equally violent at all times; like tetanus, there are paroxysms arising spontaneously, without being produced by an attempt to move any of the limbs: at other times also the rigidity remains fixed until dissolution. The circulation at first does not appear to be much affected by the disease; the pulse remains in a natural state: the same may be remarked in some cases of severe injuries of the spine; there is hardly any perceptible change until the last moments of the patient. In arachnitis, however, after a time it increases in the rapidity of its beat; it becomes quick, irritable, and feeble, but irregularity seldom or ever occurs: the tongue at first is slightly furred; as the disease advances, however, it becomes coated with a dark brown fur; it then is dry and rough, and lastly aphthous. At this period the patient usually complains of extreme thirst, with a burning sensation in the throat, and difficulty of swallowing.

"In true arachnitis, there is never paralysis of any of the limbs, or loss of sensibility in them. It often happens, however, that one or both of these symptoms occurs at the same time: when such is the case, the spinal cord itself is affected, being inflamed or softened in its structure. It is seldom found also, after the death of a patient suffering from this disease, that the arachnoid membrane is alone inflamed; the pia mater is frequently affected as well as the dura mater. The corresponding membranes of the brain also are continually inflamed, giving rise to those symptoms which usually occur in such affections of this organ.

"From the symptoms which take place in arachnitis spinalis, it may be observed that they very much resemble those of tetanus. The morbid appearances of thespine, also, are much the same in both, as I shall, when speaking on that subject, presently shew. I am inclined to think, therefore, the disease we term idiopathic, tetanus, and arachnitis, are synonymous. The only difference, if there be any, is, that arachnitis may begin in any portion of the spine, while tetanus usually makes its first appearance in the medulla oblongata; thus producing trismus, then the contraction of the muscles of the neck, and at length, opisthotonos." (P. 254.)

The treatment of this disease consists in bleeding, the application of such counter-irritants as act quickly,—the administration of purgatives. If violent spasms occur, which is frequently the case, the extract of belladonna may be rubbed along "the whole course and on each side of the spine, with the greatest advantage." Upon this point Mr. Stafford speaks from personal experience. Opiates may be necessary, but, in Mr. S.'s opinion, they should, if possible, be avoided. The resemblance between arachnitis spinalis and tetanus is illustrated by cases.

There are still a few pages of interesting remarks on different affections of the spinal marrow, but we must "grow to a point," and conclude our notice of Mr. Stafford's work by recommending it to the notice of the profession, and especially the junior members, as one which contains a clear, succinct, and practical sketch of the important diseases on which it treats.

*Two Lectures on the Primary and Secondary Treatment of Burns.* By HENRY EARLE, F.R S., Surgeon Extraordinary to the King, Surgeon to St. Bartholomew's Hospital, &c.— 8vo. pp. 59, with Plates. Longman and Co., London.

EVERY surgical student ought to peruse these lectures: they contain by far the best account we have perused of the treatment that ought to be adopted in cases of burns, together with a description of the practice originally suggested by Mr. EARLE for the purpose of remedying those deformities which so frequently result from such injuries. Mr. Earle states that numerous instances of deformity and lameness present themselves every year at St. Bartholomew's, requiring very painful and serious operations; the majority of which, he believes, might have unquestionably been prevented if the rules described in these lectures had been attended to. We shall confine the present notice to the first lecture, and of this the following is a full analysis.

Certain individuals, Mr. E. observes, appear to possess the power of resisting high temperature to a very marvellous degree; but, with the generality of mankind, heat, whether dry or moist, beyond a certain point, induces inflammation, vesication, or mortification, according to the degree of heat, the duration of its application, and probably the peculiar temperament of the individual. By this latter observation, he wishes to imply that an impaired state of the nervous influence renders the body less capable of maintaining its own standard temperature, and resisting that of surrounding media. This is the only rational explanation he can offer, and we have no doubt it is the correct one, of the very different effects produced on the human body by apparently the same extent of injury and the same degree of heat. To illustrate this the following case is mentioned.

"A lady who had been long resident in India, where she had a large family, and who had suffered considerably during her voyage home, soon after her return had the misfortune to set fire to her sleeve, which, together with part of her gown, was destroyed by the flame. She was quite dressed at the time, and her stays were only very slightly scorched on the outer side; her chemise did not appear in the least degree injured. I was called to her the day following the accident, when I found that the whole depth of skin of the entire arm, shoulder, neck, and side, down to the lower margin of the ribs, was destroyed, and had lost its vitality, the slough being deepest and longest to separate over the serratus magnus muscle, which was eventually in part exposed. Now, a very considerable part of this dead surface had not been at all exposed to the flame, the stays being only slightly scorched, but quite entire; yet the heat conveyed through them had produced such destructive effects upon the integuments. This case is certainly very remarkable; but I have no doubt that similar cases have occurred in various degrees, and I can only conjecture that the state of her nervous powers was impaired, and had in some degree lost its conservative influence. I am further borne out in this supposition by the phenomena which occur when any part of the living body is deprived, by operation or injury, of the principal supply of nervous power. Some interesting examples, illustrative of the influence of the nervous system in regulating the animal temperature, occurred in my practice many years since, which I published in the "Medico-Chirurgical Transactions," in a paper on animal heat. It may not be uninteresting to cite one or two instances briefly. I had occasion to remove a large portion of the ulnar nerve in a young female, for the cure of tic douloureux. For a long time after the removal, the parts supplied by that nerve, namely the little finger and one side of the ring-finger, were incapable of resisting changes of temperature, which did not in the least degree affect the rest of the hand. Thus, moderately hot water produced frequent vesications; and exposure to cold air induced sloughing of the extremity of the finger, and loss of the nail. In another instance, where the axillary plexus of nerves was crushed by a comminuted fracture of the clavicle, leaving the whole arm in a paralytic state, the same phenomena presented themselves. The patient, on one occasion, immersed his arm in warm grains for some time, and, on removing it, the whole hand and forearm were covered with vesications." (P. 4.)

Mr. Earle next proceeds to consider the various degrees of injury which may ensue from the application of heat, and these are arranged under three heads.

"In the first or mildest form of scald or burn, a degree only of inflammation is produced, which, by proper treatment, speedily terminates by resolution, without exciting any constitutional or symptomatic fever. Even when unassisted by art, many of these cases terminate spontaneously by resolution; occasionally, in irritable habits, such injuries will excite more febrile action, and continued sharp pain, attended with redness and swelling. In such cases vesication may follow after some interval, as a consequence of the inflammation. Under these circumstances, although the degree of injury inflicted may be moderate, the case may be of importance from the extent of surface which may have been involved, and which may render it of far greater moment than when a much greater degree of heat has been applied to a more limited surface.

"From the second form or degree of injury arising from the application of heat, whether dry or moist, vesications speedily follow, which increase in volume and number according to the nature of the substance which has conveyed the heat, and the extent to which it has been applied. You well know that different substances have different degrees of capacity for caloric; that some part with it more rapidly than others. These circumstances must be borne in mind in estimating the probable extent of injury. Thus, boiling oil will inflict a severer burn than boiling water, and boiling metal a still more severe one. In this second degree of injury from the application of heat, it commonly happens that some part of the surface is denuded of its cuticular covering, leaving a highly inflamed surface in a state of the greatest excitement, exposed to the action of the air and other stimulants. The inflammation which is excited will also terminate in more extensive vesication than what resulted from the immediate application of the heat. The constitutional disturbance consequent upon such an injury is sometimes very considerable: severe rigor, followed by fever, and much nervous excitement, commonly ensue; and if the surface injured be considerable, serious disturbance of the serous and mucous membranes not unfrequently arises from the functions of so important an organ as the skin being more or less impaired or destroyed.

"The third and most important kind of burn is that in which more or less of the integument and the more deeply seated parts are deprived of their vitality, either by the immediate violence and intensity of the heat applied, and the duration of such application, or in consequence of the high degree of inflammation which has been excited, and the peculiar temperament of the individual. Such cases are almost always combined with the two former degrees, as it very rarely happens that the whole force of the fire is expended on any given spot. One part suffers more severely, and may lose its vitality, whilst those in its neighbourhood may be vesicated and denuded, or only inflamed. Nothing can be more varied than the aspect presented by severe burns: at one part there may be an appearance of deep red, whilst others are vesicated, and a third may present the appearance of an eschar. The parts which are entirely deprived of vitality usually are of a dirty white colour, and the cuticle peels off without rising; at times they have a semi-transparent appearance; and the course of the superficial veins, filled with dry coagulated blood, may be seen crossing in different directions; around these dead portions the integuments have the deepest red, approaching to gangrene, which is gradually lost as you approach the more healthy skin: at other times, when the parts are very deeply destroyed, the eschar has a black appearance, nearly similar to the dry gangrene of the feet of old people." (P. 7.)

It is difficult, at first sight, to form any correct opinion of the extent and depth to which this destructive process may have gone: the prognosis should, therefore, be guarded when any of the above appearances are observable. The constitutional symptoms which accompany such burns are always severe.

"The extremities are generally cold, and the patient experiences rigors, which recur at irregular intervals, and are in general in proportion to the extent, and depth, and importance, of the part burnt. Exposure of the surface of the body greatly increases these rigors; so that you generally have an opportunity of witnessing this phenomenon when a patient is first admitted to the hospital. The pulse is frequent and very small. The respiration is often laborious. The stomach is irritable, and rejects its contents. Hiccough ensues, and the patient often sinks into a state of coma, in which he expires in a few hours, or after an interval of from one to two days. If the patient survive this first stage, he may fall a victim to the symptomatic fever which ensues at any period during the first fortnight. It not unfrequently happens that active inflammation of some of the serous or mucous membranes arises, which may claim all your attention, and require a plan of treatment very opposite to that which the local injury would appear to indicate. These febrile symptoms generally abate after the first fortnight; and if the case terminates unfavorably after this period, the patient sinks, from his vital powers being worn out by copious discharge and continual suffering, and he dies completely hectic. It has often occurred to me that certain days might be considered critical, from the frequency of a fatal termination on those days, particularly the third and tenth days." (P. 11.)

*Treatment of Burns.* It is obvious that no one plan of treatment can be equally applicable to such various degrees of injury as have been described. Mr. Earle first considers the different plans which have been used, and then examines the degree of credit to which they are severally entitled: he then fixes the principles which should guide the surgeon in his choice of remedial means; and, lastly, he applies these principles to the various degrees of injury, both with reference to the extent of surface injured and the intensity of the effect produced.

Two modes of treatment appear to have been employed in all ages, and these are diametrically opposed to one another; the one has for its object to diminish the inflammatory symptoms by the application of cold; the other, to effect a cure by stimulating applications. Sir James Earle was a warm advocate for the former plan, and his son "has known this plan very successfully employed in many cases, particularly of scalds, to one or more of the extremities." When the hot fluid has been directly applied to any exposed surface, as the hands, the speedy employment of cold will often prevent vesication, and the case will terminate by resolution. If the part affected is at the time enveloped in some article of clothing, as the legs and feet with stockings, more or less vesication is likely to occur, from the clothes retaining the hot liquid in contact with the skin. Under these circumstances, Mr. Earle remarks, it unfortunately happens that the first thing that is done is to remove the stocking or clothes, which never fails to bring away with it large portions of the cuticle, leaving the highly inflamed cutis quite denuded: but if, instead of this forcible removal of the clothes, such limbs were immediately immersed in the coldest water, this most serious result would generally be prevented. Should it become necessary to remove them, on account of the formation of large vesicles, they should be carefully cut away, and the vesicles preserved unbroken, by which the serious consequences which always follow the exposure of the highly inflamed cutis will be prevented. When the injury is extensive, and occurs in any part of the chest or trunk, or in a delicate constitution, cold cannot, in Mr. Earle's opinion, be employed without the risk of inducing inflammation of the pleura or peritoneum.

"The advantages which this cooling plan holds out are, that it may often be resorted to without delay, and it has the effect of affording immediate relief: the disadvantages attending it are, that it is necessary to continue and renew the application of cold for a considerable length of time, as the heat and pain will return, unless the diminished temperature be steadily maintained." (P. 16.)

In whatever manner cold is applied, the burnt part should never be exposed to the atmosphere until the inflammation be subdued, as reaction is certain to follow every such exposure.

"One of the most simple and efficacious plans is to envelop the part with rags, and to keep them constantly wetted with water, in which ice is placed from time to time; care being taken never to remove the rags from the burnt surface; whenever the vital powers are depressed, and rigors supervene, the employment of cold is prohibited, a circumstance which will occasionally happen, after even inconsiderable burns, in irritable constitutions." (P. 17.)

The contrary, or stimulating plan of treatment, is also of great antiquity. Mr. Earle says that it is now exploded, "as causing much and unnecessary pain in slighter cases, and as perfectly inadmissible in more extensive ones." Different oils and various unctuous substances have been highly extolled, and it is admitted that they are highly beneficial; but their action is referred to one common principle, which is to be kept steadily in view, viz. *as speedily as possible to exclude the air from the inflamed surface*, which never fails to stimulate and to excite an injurious degree of reaction. Flour, fuller's earth, cotton wool, &c. act in the same manner. Experience teaches us that, in more severe burns, certain medicated applications exert a very beneficial influence.

"When the object is only to exclude the air from the denuded surface, and the burn is not very severe, one of the best applications is a liniment composed of limewater and linseed oil; if fine linen be employed, well moistened with this, it will be found to answer every indication. The lime which is held in suspension completely fills up the interstices of the cloth, and effectually excludes the air; whilst the oil renders it so pliant that it may be accurately applied to every surface and cavity. The same effect may be produced with the superacetate or the carbonate of lead, and oil, where the denuded surface is not considerable. This effect of the lime or lead in closing the interstices of the linen is very similar to what occurs in the well-known experiment of immersing eggs in limewater to preserve them sweet for an indefinite time, by closing all the pores of the shell, and excluding the air." (P. 20.)

Mr. Earle grants that we are indebted to Mr. Kentish for many useful suggestions, but to his theory he offers very reasonable objections.

"The practice which I have been long in the habit of pursuing, with very happy results, has been to bathe the parts with warm spirits of turpentine; and, as speedily as possible, to envelope every part most carefully with soft lint, thickly spread with the liniment of turpentine and resin cerate. It is better entirely to surround the extremities when burnt, and to retain the dressings with bandages, accurately but not too tightly applied. This application appears very soothing; and the young patient will often cease to cry, and will even fall asleep as soon as dressed.

"Having very accurately covered every part of the burnt surface with the dressing, the patient should be suffered to remain quiet, and the dressings should not be disturbed for many days; not, indeed, until suppuration is fully established. If, on removing the dressings, deep sloughs present themselves, there is no better application than warm emollient poultices. After the separation of the sloughs, the wounds must be dressed, and treated like common ulcers; but it is not my intention, at the present moment, to enter upon the subject of the secondary treatment of burns. The same rules with respect to the mode and accuracy of dressing burns should be adhered to, whether we employ Kentish's ointment, or any other application. The principle of excluding the air and not unnecessarily disturbing the patient should be steadily kept in view, whatever may be the remedial means resorted to." (P. 23.)

*Constitutional Treatment of recent Burns.* It is generally requisite, after the receipt of any considerable burn, to administer some cordial internally, combined with opiates. If the pulse is small and feeble, extremities cold, and a disposition to rigors, warm brandy or wine and water, or ammonia with laudanum, according to the age of the patient, should be given. If the vital powers are not depressed, and the patient suffers much, opium may be given without the spirit. Mr. E. remarks, that considerable judgment is required in administering cordials, and upon this subject he offers some very sensible comments, in answer to Mr. Kentish's doctrines. Mr. K. advises the giving powerful stimuli internally, on the principle of counter-irritation, and he advocates the perseverance in their use for several days, until secretion has taken place. It is true, says Mr. E., that he adduced some strong facts in illustration of this plan; "but I could produce equally powerful arguments to prove that such a practice is most injurious and directly opposed to the dictates of common sense, and all the principles laid down for the treatment of inflammation. To adduce one memorable instance in which this stimulating plan was most injuriously persevered in, I will mention the cases of the firemen who suffered at the burning of Covent Garden theatre. Several of these unfortunate men were admitted into this hospital, and died with every symptom of inflammation of the membranes of the brain and mucous linings of the lungs." (P. 25.)

The stimulating plan was carried too far in these cases, which was fully proved by examination after death.

"If the vital powers be greatly depressed, you may administer a cordial, and even repeat it until reaction has taken place; but when once that has occurred, it can rarely, if ever, be necessary to persevere in such a plan. When the first stage is passed, light and nutritious farinaceous food should be given, and the bowels gently regulated: opiates will often be required for some time, to allay the irritation and pain. Diarrhœa sometimes supervenes, and is occasionally beneficial when the discharge is very copious. From observing the occasional good effects resulting from spontaneous diarrhœa, when the discharge was profuse, in accelerating the healing process, Mr. Kentish strongly recommends the free use of purgatives under such circumstances, and I have known them very useful. The spontaneous diarrhœa, however, requires to be very carefully watched, as it may arise from a destructive inflammation of the mucous membrane of the bowels, which may require all our efforts to control." (P. 26.)

In the application of cold to burns, some caution is necessary. When the surface is considerable, including any part of the trunk, there would be danger in continuing it, though Mr. E. thinks it would be quite justifiable to plunge a person with an extensive scald, with all his clothes on, into cold water; but he cautions the practitioner against the danger of inducing inflammation of the serous or mucous membrane of the chest and abdomen, by the continuance of it.

"This circumstance should indeed be always carefully borne in mind, whatever be the treatment employed, as one very likely to occur in extensive burns, from the impaired functions of the integuments of the body, by which an additional burden is thrown on the lungs especially. You are well aware that, in a state of health, there is a constant transpiration going on from the whole surface. When this is checked by sudden cold, pneumonia, pleurisy, or peritonitis, not unfrequently ensue. Exactly the same effect is produced by extensive burns or scalds, which necessarily interfere with this most important function performed by the skin. When you have reason to suspect any of these consequences, bleeding, both local and general, may be required: but, generally speaking, salines, particularly small and repeated doses of nitrate of potash, by increasing the action of the kidneys, will render this unnecessary."

When there is more or less of the cuticle destroyed, and the highly inflamed cutis is exposed, it is right to exclude the air as soon as possible; and, for this purpose, limewater and linseed oil is recommended as a very efficacious remedy. If the vesications are large and in danger of bursting, they should be punctured with a needle at several points in the direction of the scales of the cuticle.

In the third order of burns, Mr. Earle knows of no plan superior to Mr. Kentish's, but he would modify his treatment in the after stages, and on no account follow it up day after day, under the visionary and erroneous impressions Mr. K. entertained. It is indifferent what application is made to parts whose vitality is destroyed. It is to the parts in the immediate vicinity, those which are in the highest state of inflammation, bordering on sphacelation, that attention must be directed; and, from extensive experience, Mr. E. affirms that the employment of the stimulating dressings of Kentish will be found preferable to any other plan.

In our next number we shall notice the second lecture; and we cannot now conclude without complimenting Mr. Earle for the clear and satisfactory manner in which he has laid down the practice to be followed in cases of the injuries he mentions.

